# The potential for parasite spill-back from commercial bumblebee colonies: a neglected threat to wild bees?

**DOI:** 10.1007/s10841-021-00322-x

**Published:** 2021-05-22

**Authors:** Callum D. Martin, Michelle T. Fountain, Mark J. F. Brown

**Affiliations:** 1 Centre for Ecology, Evolution and Behaviour, Department of Biological Sciences, Royal Holloway, University of London, Egham, Surrey TW20 0EX UK; 2 East Malling Research, NIAB EMR, East Malling, Kent, ME19 6BJ UK

**Keywords:** Commercial bumblebee management, Parasite spill-over, Wild bee conservation, Strawberry crop, Pollination

## Abstract

Commercially-reared bumblebee colonies provide pollination services to numerous crop species globally. These colonies may harbour parasites which can spill-over to wild bee species. However, the potential for parasites to spread from wild to commercial bumblebees, which could then lead to parasite spill-back, is poorly understood. To investigate this, parasite-free commercial *Bombus terrestris audax* colonies, which are used commercially for strawberry pollination, were placed into seasonal strawberry crops for either 6- or 8-week blocks across two key time periods, early spring and early summer. Bumblebees were removed from colonies weekly and screened for the presence of parasites. In the early spring placement, only one parasite, the highly virulent neogregarine *Apicystis bombi*, was detected at a low prevalence (0.46% across all bees screened). In contrast, all colonies placed in the crop in the early summer became infected. A trypanosome, *Crithidia bombi*, and *A. bombi* were the most prevalent parasites across all samples, reaching peak prevalence in screened bees of 39.39% and 18.18% respectively at the end of the experimental period. The prevalence of *A. bombi* was greater than most UK records from wild bumblebees, suggesting that commercial colonies could enhance levels of *A. bombi* infection in wild bees through spill-back. Studies on larger geographical scales with different commercial colony densities are required to fully assess spill-back risk. However, seasonal management, to minimise spill-back opportunities, and treatment of commercial colonies to prevent infection, could be implemented to manage the potential risks of parasite spill-back to wild bees.

**Implications for insect conservation** Our results show that commercial bumblebee populations do pick up infections, most likely from wild bees, and that these infections can reach prevalences where they may pose a threat to wild bees via parasite spill-back. More research is required to clarify the extent of this potential threat.

**Supplementary Information:**

The online version contains supplementary material available at 10.1007/s10841-021-00322-x.

## Introduction

Commercial bumblebees have been produced and used for crop pollination since the 1980s (Velthuis and van Doorn 2006). They were first commercialised for the pollination of greenhouse tomato plants and the success of this has led to them being used on a wide variety of crop types (Velthuis and van Doorn 2006). The trade in commercial colonies is now a global industry with over 1 million colonies shipped around the world, and 40–50 thousand imported to the UK annually (Velthuis and van Doorn 2006; Natural England 2009, 2012).

While commercial bumblebees provide significant pollination services to commercial crops (Morandin et al. 2001a, 2001b; Roldán Serrano and Guerra-Sanz 2006; Martin et al. 2019), there are several drawbacks to their use. Firstly, commercial bumblebees can breed with native species (Kanbe et al. 2008; Kondo et al. 2009), which can potentially contribute to biodiversity homogenisation and introgression of maladaptive alleles in native populations (Meeus et al. 2011; Seabra et al. 2019). Secondly, commercial bumblebees are able to outcompete native species for resources (Ings et al. 2006; Inoue et al. 2008). Finally, commercial colonies can harbour pathogens (Graystock et al. 2013), and previous studies found evidence that when commercial colonies were placed in crops, pathogen spill-over to wild bee populations occurred (Colla et al. 2006; Goka et al. 2006; Murray et al. 2013). The most likely mechanisms of transmission are via the flowers of the target crop, which both commercial and wild bees may visit, and via the well-documented occurrence of commercial bumblebees leaving the target crop and foraging on alternative resources where wild bees also forage (Murray et al. 2013; Foulis and Goulson 2014). Such pathogen spill-over is thought to be one of the contributing factors to declines in bumblebee species in the US and South America (Cameron et al. 2011, 2016; Meeus et al. 2011; Schmid-Hempel et al. 2014).

Pathogen spill-over from commercial bumblebee colonies to wild bees has been relatively well studied (see references above), and has led to changes in commercial rearing to eliminate pathogens from their colonies (Department for Environment Food and Rural Affairs 2018), but little consideration has been given to pathogen transmission in the other direction in this system. Studies of parasite-free lab reared colonies placed outside into the field, suggest that transmission from wild bees is very likely to happen (Imhoof and Schmid-Hempel 1999; Baer and Schmid-Hempel 2001).

Commercial bumblebee colonies can occur at high densities on farms (Whitehorn et al. 2013). If these colonies become infected with a parasite from an external source, subsequent transmission of the parasite between colonies could occur rapidly, leading to high parasite prevalences in commercial bee populations. These populations could then act as sources for further infection of wild bees. This process is known as ‘spill-back’. Spill-back occurs when a non-indigenous species or population can host and amplify the prevalence of an indigenous parasite species. The non-indigenous host can then act as a source for re-infection of the indigenous host (Kelly et al. 2009). Such spill-back has been documented in other systems and is thought to have the potential to negatively impact upon indigenous wildlife populations (Kelly et al. 2009; Nugent 2011). The potential for spill-back to occur in a commercial bumblebee-wild bee system is not known. Given that bumblebee parasites are known to reduce the fitness and alter the behaviour of their hosts (Brown et al. 2000, 2003; Gegear et al. 2006; Otti and Schmid-Hempel 2007; Graystock et al. 2016), not only could spill-back be harmful to native bee populations, but it could also negatively impact upon commercial colonies with subsequent implications for crop pollination and agricultural outputs.

A factor that might enhance the susceptibility of commercial bumblebees to spill-back is the large geographic scale over which this trade occurs. Colonies are often shipped hundreds or thousands of miles to reach their target crop (Velthuis and van Doorn 2006), meaning that they may not have previously encountered the parasite species and strains that the local bee populations are carrying. Novel strains from different geographical areas can induce higher mortality than strains that the population has co-evolved with (Imhoof and Schmid-Hempel 1998a), resulting in higher impacts on commercial colonies than those faced by the native bee population. In addition, parasites may reproduce to higher levels in these naïve or non-adapted commercial hosts, which would enhance the potential for spill-back.

The time of year in which commercial bumblebees are deployed on a crop is also likely to have an effect on the likelihood of colonies spreading parasites to, or picking up parasites from, wild bee populations. Crops grown in polytunnels and greenhouses can have extended flowering periods, including at times when wild bumblebees are not abundant in temperate regions. Commercial bumblebees deployed at these times are less likely to encounter wild bees or flowers recently visited by wild bees, and are thus less likely to transmit or become infected with pathogens. In addition, commercial colonies placed in crops early in the season may not be able to forage outside at all, as greenhouses and polytunnels may be completely sealed. This mismatch between commercial bee use and wild bee foraging activity may not apply in tropical regions where conditions are sufficient for year round bee foraging.

To assess the potential for pathogen spill-back to occur from commercial bees, we first need to know the pathogen dynamics in these colonies when they are placed into crop systems. Studies have shown that commercial colonies can carry parasites (Whitehorn et al. 2013; Murray et al. 2013; Graystock et al. 2013; Sachman-Ruiz et al. 2015; but note that substantial work has been done to remove parasites from commercial stock, see above), but we currently know little about the dynamics of parasite prevalence in commercial colonies during their time in a crop. Previous research suggested that parasite prevalences can become high in these systems (Whitehorn et al. 2013), and that a high initial level of parasitism can be maintained in commercial colony systems (Trillo et al. 2019). In such cases commercial colonies could pose a threat to wild bees. However, it is not known if commercial colonies regularly pick up parasitic infections from wild bees, and whether these parasites are able to develop high prevalences in commercial bumblebee populations in a farm setting.

In this study, we screened commercial bumblebees from colonies on a strawberry farm for parasites throughout a 4-month period of the growing season to address the following questions: (1) do commercial bumblebee colonies acquire parasites from wild populations? (2) If so, do these infections develop to levels that could potentially pose a risk to wild bees via spill-back? and (3) does the time of year that commercial bumblebees are deployed affect their likelihood of acquiring a parasitic infection and developing high prevalence infections?

## Methodology

The field site, spatial and temporal colony arrangements, and study species are described in detail in Martin et al. (2019). Here we give an abridged version of those aspects of the methodology.

The field site was an 80-hectare soft fruit farm in Kent, in the South East of the United Kingdom (latitude: 51.288694, longitude: 1.183766). On 21 March 2016, nine commercially reared (Biobest) *Bombus terrestris audax* colonies were placed across three fields planted with a June-bearing strawberry crop. These colonies remained in the crop for six weeks until being removed on 29 April. On 9 May, a second set of twelve commercially reared colonies were placed across a different set of four fields of an everbearing strawberry crop. These colonies remained in the crop for eight weeks, being removed on 1 July. These time periods were chosen as they coincided with the peak flowering period of the strawberry crops and the commercial bumblebee suppliers recommended that colonies remain in crops for six to eight weeks. The colonies were opened and closed on a weekly schedule as part of the data collection process of a separate experiment (detailed in Martin et al. 2019).

In the June-bearing strawberry crops, the mean density of colonies across the three fields was 2.83 colonies ha^−1^, and in the everbearing strawberry crop the mean density across the four fields was 1.52 colonies ha^−1^. These densities are different to those reported in Martin et al. (2019) as for the purpose of this experiment, the colony densities were calculated over entire fields that colonies were placed in.

All strawberry crops were grown on an elevated table-top system in polytunnels. In the June- and everbearing crops, the ends of the polytunnels were open, allowing bees to leave and enter the polytunnels. In addition, in the everbearing crop, the sides of the polytunnels were rolled up. All bumblebee deployment and polytunnel practices followed standard management approaches.

### Bumblebee sampling

Bumblebees were removed from commercial colonies using metal forceps. The forceps were submerged in alcohol and flamed between sampling from different colonies to avoid transmission of pathogens via this route. To determine initial levels of infection in commercial colonies, 10 bees were removed from each colony immediately after colonies were delivered to the field site, and before colonies were opened, so there was no possibility that an infection could have been picked up from an external source at this time point. After this initial sample, bees were positioned in the strawberry crop and sampled at the end of each week of the experiment. Initially, 10 bees were sampled from each colony, but after the first week of the June-bearer experiment this was reduced to 5 to avoid large decreases in colony populations that might impinge on normal behaviour. During week 25, colony EV3 was not sampled due to a sampling error made by the experimenter (see Supplementary Table S1b). Upon removal from the colonies, all bees were placed into plastic vials and temporarily stored, for between one to four days, in a freezer at approximately − 15 °C, before being transferred to a − 80 °C freezer for longer term storage. At the end of the final week of both experiments, colonies were closed at nightfall, to ensure almost all workers were inside, and placed into a − 20 °C freezer. Once the bees were freeze killed, a further 10 bees from each colony were collected and placed into the − 80 °C freezer for later dissection.

### Dissection

The abdomens of all sampled bumblebees were dissected and examined for the presence of conopid fly larvae (*Conopidae*) and tracheal mites (*Locustacarus buchneri*). The hind gut, Malpighian tubules and fat body were removed from the abdomen and individually examined under a phase-contrast microscope at 400× magnification for the presence of the parasites *Crithidia bombi*, *Nosema bombi* and *Apicystis bombi* (Rutrecht and Brown 2008).

### Statistical analyses

Statistical analyses were performed using R programming software (R Core Team 2018). Overall parasite prevalence (the prevalence of infection with at least one of the investigated parasite species), *C. bombi* prevalence and *A. bombi* prevalence were all analysed using binomial mixed effects models in the package ‘lme4’ (Bates et al. 2017). Calendar week was fitted as a fixed effect, and colony identity was fitted as a random effect. For the overall parasite prevalence model ‘sampling event’ was fitted as an observation-level random effect to account for overdispersion (Harrison 2015). The proportion of colonies infected with all parasites, and individually with *C. bombi* and *A. bombi* were analysed using binomial general linear models, with calendar week as the only fixed effect. Models were validated by visual inspection of the residuals plotted against the fitted values. Models were not overdispersed.

The overall parasite prevalences in the colonies from the June- and everbearing crops were compared using a two-sample z-test for equality of proportions.

**Fig. 1:**
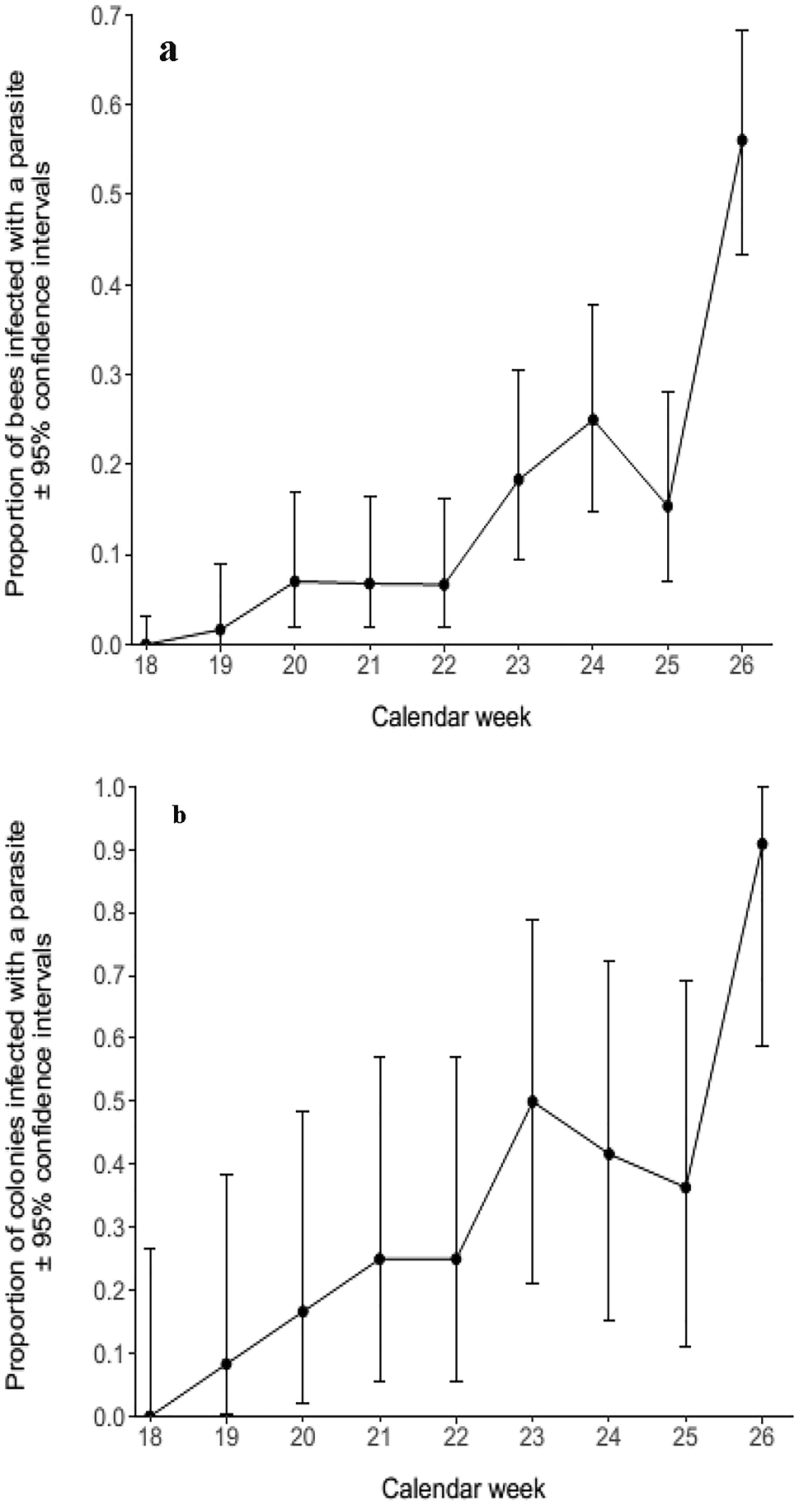
**a** The proportion of *B. terrestris* from the commercial colonies placed in the everbearing strawberry crop that were infected with at least one parasite species. **b** The proportion of colonies placed in the everbearing strawberry crop that were infected with at least one parasite species

**Fig. 2:**
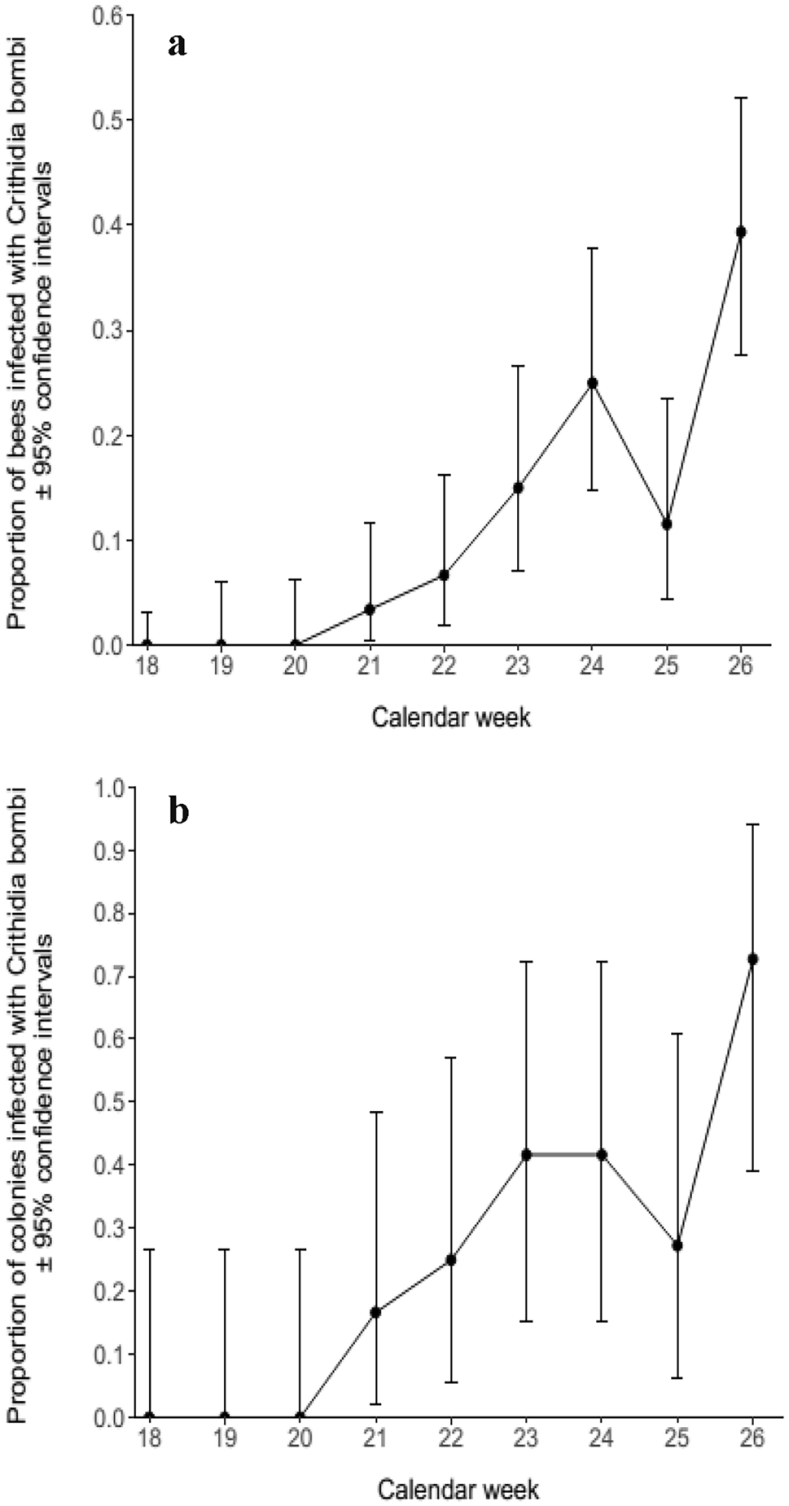
**a** The proportion of *B. terrestris* from the commercial colonies placed in the everbearing strawberry crop that were infected with *C. bombi*. **b** The proportion of colonies placed in the everbearing strawberry crop that were infected with *C. bombi*

**Fig. 3:**
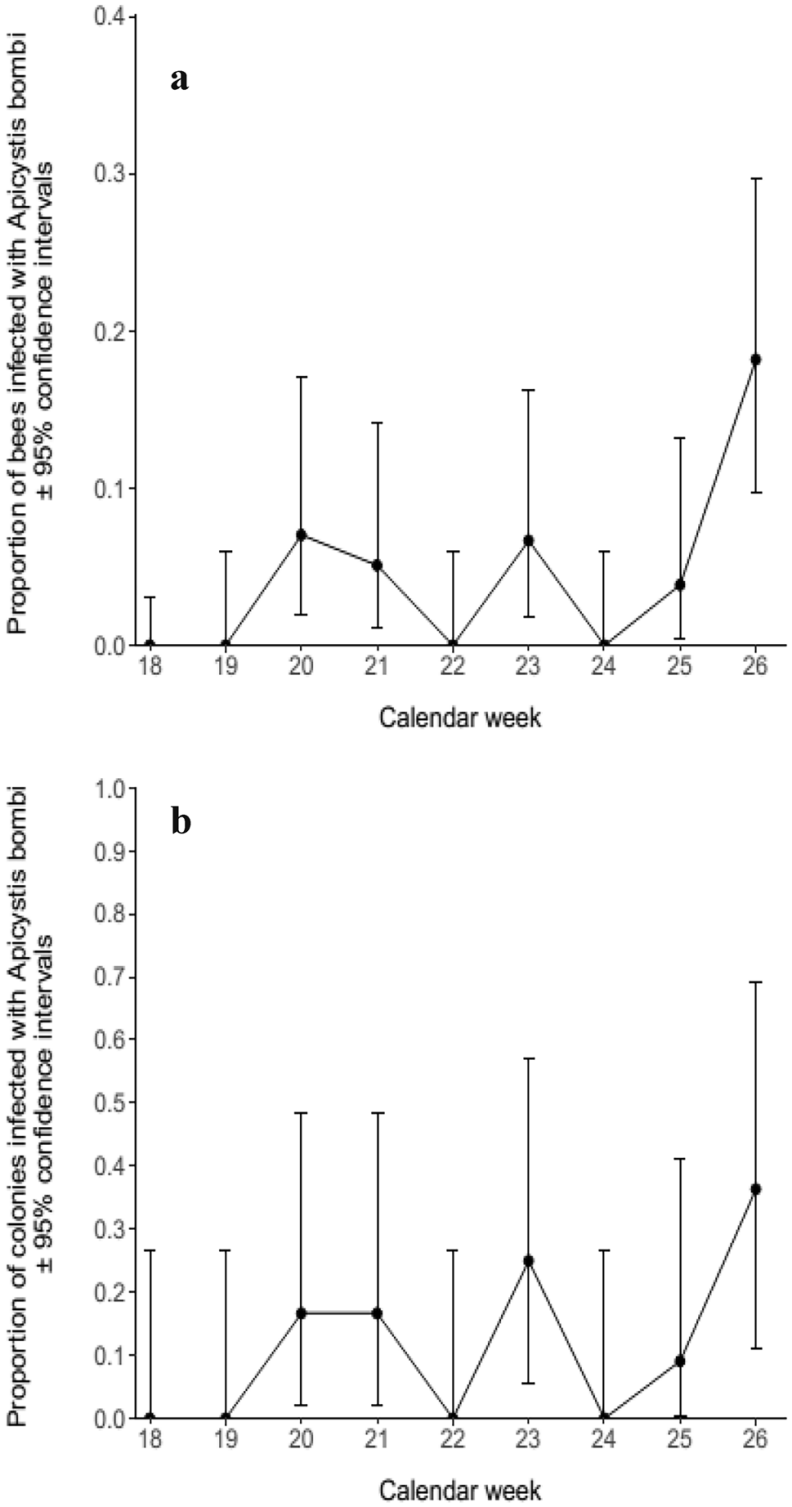
**a **The proportion of *B. terrestris* from the commercial colonies placed in the everbearing strawberry crop that were infected with *A. bombi*. **b** The proportion of colonies placed in the everbearing strawberry crop that were infected with *A. bombi*

95% binomial proportion confidence intervals were calculated around all prevalence estimates using the Clopper–Pearson ‘exact’ method (Clopper and Pearson 1934), and these intervals are presented in Figs. 1, 2 and 3.

## Results

### Parasite prevalence

A total of 438 bumblebees were dissected from the nine colonies placed in the June-bearing strawberry crop (see Supplementary Table S1a for number of bees dissected each week). Here, and in the everbearing crop trial, the planned number of bees could not be sampled from all colonies, due to colony senescence. No parasites were detected in the initial screen, indicating that colonies were likely free of the parasite species we screened for on placement into the crop. It is possible that parasite species could have gone undetected by our sampling method.

The only parasite observed in the June-bearer colonies was *A. bombi*, which was detected in two bees {a prevalence of 0.46% [95% confidence interval (CI): 0.055–1.64%]} from a single colony from the final sampling time point of the experiment.

594 bumblebees were dissected from the twelve colonies placed in the everbearing crop (see Supplementary Table S1b for number of bees dissected each week). Again, no parasites were detected in the initial screen, indicating that colonies were likely parasite-free on placement. The overall parasite prevalence for the duration of the experiment was 14.14% (95% CI: 11.44–17.21%), which was significantly higher than the overall prevalence from the June-bearer colonies (z-test for equality of proportions: X^2^ = 60.03, *p* < 0.05). *C. bombi* was the most prevalent parasite throughout the experiment at 10.44% (95% CI: 8.10–13.18%), followed by *A. bombi* at 4.21% (95% CI: 2.74–6.15%). Eleven colonies became infected with *C. bombi* at some point during the experiment with six of these colonies also acquiring *A. bombi*. Only one colony was solely infected with *A. bombi* (Supplementary Table S1b). Conopid fly larvae (Conopidae) were only detected in three bees from three colonies from the final week of the experiment. *Nosema bombi* was detected in only one bee from calendar week 19. Conopid fly larvae and *N. bombi* were not analysed further because of the low prevalence.

Overall parasite prevalence increased during the everbearer experiment and a particularly large increase in prevalence occurred during the final week of the experiment (Fig. 1a), where a peak prevalence of 56.06% (95% CI: 43.30–68.26%) was reached. The proportion of colonies infected by a parasite also increased throughout the experiment, and by the final week 90.91% (95% CI: 58.72–99.77%) of colonies were infected by at least one parasite species (Fig. 1b). The calendar week was a significant predictor of overall parasite prevalence (GLMM: *z* = 6.40, *p* < 0.05), as well as the proportion of colonies infected by a parasite (GLM: *z* = 4.42, *p* < 0.05) in the everbearing crop.

*Crithidia bombi* prevalence across all samples was 10.44% (95% CI: 8.10–13.18%), but it had a pronounced temporal dynamic, and reached a high of 39.39% (95% CI: 27.58–52.19%) during the final week of the experiment, after displaying a general trend of increasing prevalence during the experiment (Fig. 2a). The proportion of colonies infected by *C. bombi* also increased throughout the experiment (Fig. 2b). By the final week 72.73% (95% CI: 39.03–93.98%) of colonies were infected with the parasite. The calendar week was a significant predictor of both *C. bombi* prevalence (GLMM: *z* = 7.37, *p* < 0.05) and the proportion of colonies infected by *C. bombi* (GLM: *z* = 4.30, *p* < 0.05).

*Apicystis bombi* was detected at a prevalence of 4.21% (95% CI: 2.74–6.15%) across all samples from the colonies placed in the everbearing crop. It reached a peak prevalence of 18.18% (95% CI: 9.76–29.61%) in the final week of the experiment (Fig. 3a). The proportion of colonies infected with *A. bombi* displayed a trend for increasing throughout the experiment, with a peak of 36.36% (95% CI: 10.93–69.21%) colonies being infected by the end of the experiment (Fig. 3b). The calendar week was again a significant predictor of *A. bombi* prevalence (GLMM: *z* = 3.87, *p* < 0.05), but not of the proportion of colonies infected by *A. bombi* (GLM: *z* = 1.92, *p* > 0.05).

## Discussion

In this study, we demonstrated that commercial *Bombus terrestris audax* colonies placed into an early summer strawberry crop became infected with parasites *A. bombi* and *C. bombi*, that were likely to have been acquired from wild bee populations. These parasites increased in prevalence in the commercial bee population through May and June, and could potentially spill-back to wild bee populations. In the case of the neogregarine *A. bombi*, the peak prevalence reached was higher than most other UK records, suggesting that it could have the potential to pose a hazard to wild bee populations if it were to spill-back to these populations. However, the presence and prevalence of parasites were highly dependent on the time of year that the commercial colonies were deployed, with colonies that were deployed earlier in the growing season (March) being significantly less likely to become infected than those deployed in May.

Parasites were detected in all twelve colonies that were placed in the crop during May and June. Our screening of colonies prior to placement in the field indicated that they were free of the parasites screened for. That commercial colonies used in this experiment were free of these parasites matches our experience with commercial colonies kept enclosed in the lab throughout their lifespan over the last 5 years (~ 250 colonies, MJFB unpublished data). Thus, this strongly supports the conclusion that all the parasites found in crop-deployed colonies were contracted from wild bees. The calendar week was a strong predictor of parasite prevalence with a clear increase in prevalence during the time that the colonies were in the crop. A peak parasite prevalence of 56.06% was reached at the final sampling time point, with 10 out of 11 colonies (one colony could not be sampled at the final time point, but this colony had been infected with *C. bombi* for the previous 4 weeks) being infected with either *C. bombi* or *A. bombi*. Individually, *C. bombi* and *A. bombi* both also reached their peak prevalences during the final week.

For *C. bombi* the general increase in prevalence during the late spring and early summer is comparable to patterns in wild bumblebee populations, which are thought to be due to the growth in bumblebee populations causing an increase in the rate of transmission of the parasite (Imhoof and Schmid-Hempel 1999; Gillespie 2010; Popp et al. 2012). During the final week of the experiment, *C. bombi* displayed a marked increase in prevalence of 27.86% reaching its peak prevalence of 39.39% and infecting 8 out of 11 colonies. Although dramatic, such an increase in a short space of time is not unprecedented (see Korner and Schmid-Hempel 2005). A study by Whitehorn et al. (2013) observed a similar pattern of *C. bombi* prevalence in *B. terrestris* populations on soft fruit farms that deployed commercial bumblebees. They also observed a marked increase in prevalence late in the season. However, they could not determine whether the bees they sampled were commercial or wild, which is essential for assessing the potential for parasite spill-back. The prevalence of *C. bombi* recorded for the whole everbearer experiment was 10.44%; this is lower than most *C. bombi* prevalences recorded in wild UK bumblebee populations (Whitehorn et al. 2011, 2013; Goulson et al. 2012; Graystock et al. 2014) and in populations from Europe and North America (Shykoff and Schmid-Hempel 1991; Gillespie 2010; Popp et al. 2012). Some studies have found prevalences similar to or lower than those found here, but these studies sampled newly emerged queens in spring (Rutrecht and Brown 2008; Jones and Brown 2014), a time when *C. bombi* is normally at its lowest prevalence. The peak prevalence of 39.39% is closer to the prevalences previously recorded for *C. bombi* in summer, but still lower than many. Based only on parasite prevalence, these results suggest that, at these in-crop commercial colony densities, *C. bombi* transmission may not pose an additional risk to wild populations. However, these results should be treated with caution, as factors such as the parasite load of commercial bees and the parasite prevalence in the wild bee population at the field site were not recorded as part of this study. Such factors play an important role in the true risk that parasite spill-back poses. It should be noted that most other studies recording *C. bombi* prevalence sample foraging bees outside the nest, rather than taking bees from directly within the nest as in this study. Such differences in sampling methodology may impact on parasite prevalence estimates.

At 18.18%, the peak prevalence of *A. bombi* was higher than most other records from wild populations in the UK and Ireland (Rutrecht and Brown 2008; Goulson et al. 2012; Jones and Brown 2014). One study from the UK did find a higher prevalence (Graystock et al. 2014), but this study only used molecular screening to detect parasites and thus cannot be certain that all detected parasites were true infections rather than bees carrying non-infecting parasite stages in their guts. In this study, we can be sure of the infection status of individual bees. As there is evidence that *A. bombi* may be highly virulent (Rutrecht and Brown 2008; Jones and Brown 2014; Graystock et al. 2016), it is of concern that this parasite can be acquired by commercial colonies and proliferate to a high prevalence within them, even at the relatively low colony densities used in this study. Subsequent spill-back of *A. bombi* to wild bumblebees could pose a threat to their populations. However, as with *C. bombi*, parasite prevalence in the wild population and parasite load in the commercial population are important factors to consider when assessing spill-back risk. Further studies are required to assess the risk spill-back could pose.

Within individual colonies, *A. bombi* was detected sporadically; after its first detection in a colony it was often not detected again for several weeks or at all. One explanation for this is that the colonies are acquiring the infection and then quickly clearing it, however, not enough is known about the epidemiology of *A. bombi* infections in bumblebee colonies to comment on the likelihood of such an occurrence. It is also possible that the mortality induced by the parasite makes *A. bombi* infected bees less likely to be sampled. We believe the most likely explanation is that *A. bombi* is generally found at low prevalences (Rutrecht and Brown 2008; Goulson et al. 2012; Jones and Brown 2014), meaning that we were unlikely to detect it within a colony every week given the sample sizes available. *C. bombi* was also detected sporadically within colonies, although not to the same extent as *A. bombi*. Bumblebees can clear *C. bombi* infections, albeit under artificial conditions (Imhoof and Schmid-Hempel 1998b), but it is likely that the number of bees being sampled was the major contributor to this detection pattern, particularly in the weeks preceding the final week, where its population prevalence rarely exceeded 20%. That the more prevalent parasite was less sporadic in its detection also supports the idea that *A. bombi* was not cleared, and remained present in colonies after its initial detection.

Given that sample sizes were fixed at either 5 or 10 bees per week, fluctuation in the size of the commercial colonies will have impacted the likelihood of detecting infections at our sampling time points. This may have been particularly evident toward the end of the everbearer strawberry experiment, where colony populations were known to be reaching relatively low levels. Thus, the number of bees sampled will have been a higher proportion of the total colony population, and consequently, these samples may be more representative of the true parasite prevalence. Taking this into account, it is possible that our estimated parasite prevalences, prior to the final week of the everbearer experiment are conservative. In the shorter duration June-bearer experiment, colonies were unlikely to have declined to the same extent, thus changes in parasite detection probability were likely not as extreme.

In contrast with the colonies used during May and June, those deployed in March on the June-bearing strawberry crop displayed low levels of parasitism. The only parasite observed in the June-bearer colonies was *A. bombi*, which was detected in two bumblebees from a single colony at the final sampling time point. These very low observed parasite prevalences are most likely due to the time of year that these colonies were deployed. In the south east of England during March and April, queen bumblebees are still emerging from hibernation and if colonies have been established, they are in the early stages of development with only very small numbers of foraging workers (Sladen 1912). This means bee abundance in the environment is low, and thus, the chances of parasite transmission between bees is greatly reduced. Furthermore, *C. bombi* prevalences are generally much lower in the early stages of the colony life cycle, usually reaching peak levels in the summer months (Imhoof and Schmid-Hempel 1999; Gillespie 2010; Popp et al. 2012). Thus, there is less chance that any bees that are contacted are carrying an infection. In the June-bearer crop, the plastic on the sides of the polytunnels was rolled down (the ends remained open), potentially making it more difficult for commercial bees to leave the crop. In addition, less clement weather and potentially less wild forage may reduce the probability of commercial bees leaving the crop. Together, these patterns are likely to explain the almost zero levels of parasitism we observed in the commercial colonies placed in the crop during March and April, and are indicative of an almost complete lack of potential for parasite spill-back. These results suggest that commercial bumblebees deployed in the UK during this time are unlikely to acquire parasites from wild bees and are subsequently unlikely to pose a threat to wild bees via parasite spill-back. Further studies over larger geographical areas would be required to confirm this.

We clearly show that commercial bumblebee populations do pick up infections, most likely from wild bees, in a commercial farm setting, and that these infections can reach prevalences similar to or, in the case of *A. bombi*, greater than those found in the wild. This suggests that there may be potential for parasite spill-back to occur from commercial colonies to wild bees. We also show the importance of time of year on the prevalence of parasites in commercial bee colonies, information that is useful for mitigating any potential environmental damage from commercial colonies. This study was done on one farm in the South-East of the UK. Spill-back dynamics could depend on location specific factors (e.g. the wild bee community present). Thus, it is difficult to extrapolate our results over a large geographical area. However, we do highlight that there is potential for parasite spill-back to pose a risk to wild bees. Further studies on farms in different locations, on different crops and with different bee densities are necessary to fully understand the potential of commercial colonies as threats to wild bees via parasite spill-back.

However, our results have possible management implications for the use of commercial bumblebee colonies for pollination. First, the risks of pathogen spill-back are likely to vary with season, and thus use of commercial colonies should reflect the phenology of wild bees to minimise interaction and hence pathogen transmission. Given that the pollination value of commercial colonies may vary in parallel ways (Martin et al. 2019), this could present a financial gain to farmers. Second, if commercial production has eliminated pathogens from commercial colonies prior to placement, as our results suggest, prophylactic medication could be used to prevent infection from wild bees. Recent studies have demonstrated the value of plant secondary chemicals in controlling or preventing infection in bumblebees (Manson et al. 2010; Richardson et al. 2015; Koch et al. 2019; Folly et al. 2020), and medication for commercial colonies is thus an obvious management step to prevent pathogen spill-back. Commercial bumblebee colonies provide important pollination services to valuable crops globally. Thus, appropriate management can play a significant role in minimising the potential costs they impose on wild bees.

## Supplementary Information

Below is the link to the electronic supplementary material.

Supplementary Table S1 (DOCX 19 kb)

## Data Availability

The data collected and analysed for this study is available to download at the following link: https://figshare.com/articles/dataset/Martin_et_al_2021_Journal_of_Insect_Conservation_xlsx/14601417.

## Code availability

The R code used to analyse the data for this study is available from the corresponding author upon request.
