# Supplementary material for: The potential for parasite spill-back from commercial bumblebee colonies: a neglected threat to wild bees?
Source: J Insect Conserv. 2021 May 22;25(3):531–9. doi: 10.1007/s10841-021-00322-x (PMC8550768; doi:10.1007/s10841-021-00322-x)
Supplement: Supplementary file 1 — Supplementary Table S1 (DOCX 19 kb) [file 10841_2021_322_MOESM1_ESM.docx]

**Supplementary Table S1.** *Infection dynamics of* Crithidia bombi *and* Apicystis bombi *in each colony used in the* ***(a)*** *June-bearing strawberry crop and* ***(b)*** *everbearing strawberry crop.* *Small numbers indicate the number of bees that were dissected from each colony at each time point.*

**a**

|  |  |  | **Calendar week** | | | |  |
| --- | --- | --- | --- | --- | --- | --- | --- |
| **Colony** | **11** | **12** | **13** | **14** | **15** | **16** | **17** |
| **JB1** | **10** | **10** | **5** | **5** | **5** | **5** | **10** |
| **JB2** | **10** | **10** | **5** | **5** | **5** | **5** | **10** |
| **JB3** | **10** | **10** | **5** | **5** | **5** | **5** | **10** |
| **JB4** | **10** | **10** | **5** | **5** | **5** | **5** |  |
| **JB5** | **8** | **10** | **5** | **5** | **5** | **5** | **10** |
| **JB6** | **10** | **10** | **5** | **5** | **5** | **5** | **10** |
| **JB7** | **10** | **10** | **5** | **5** | **5** | **5** | **10** |
| **JB8** | **10** | **10** | **5** | **5** | **5** | **5** | **10** |
| **JB9** | **10** | **10** | **5** | **5** | **5** | **5** | **10** |
| **Total** | **88** | **90** | **45** | **45** | **45** | **45** | **80** |

**b**

|  |  |  | **Calendar week** | | | |  |  |  |
| --- | --- | --- | --- | --- | --- | --- | --- | --- | --- |
| **Colony** | **18** | **19** | **20** | **21** | **22** | **23** | **24** | **25** | **26** |
| **EV1** | **10** | **5** | **5** | **4** | **5** | **5** | **5** | **2** | **1** |
| **EV2** | **10** | **5** | **5** | **5** | **5** | **5** | **5** | **5** | **10** |
| **EV3** | **10** | **5** | **5** | **5** | **5** | **5** | **5** |  | **4** |
| **EV4** | **10** | **5** | **5** | **5** | **5** | **5** | **5** | **5** | **1** |
| **EV5** | **10** | **5** | **5** | **5** | **5** | **5** | **5** | **5** | **10** |
| **EV6** | **10** | **5** | **5** | **5** | **5** | **5** | **5** | **5** | **10** |
| **EV7** | **10** | **5** | **5** | **5** | **5** | **5** | **5** | **5** | **2** |
| **EV8** | **10** | **5** | **5** | **5** | **5** | **5** | **5** | **5** |  |
| **EV9** | **10** | **5** | **5** | **5** | **5** | **5** | **5** | **5** | **7** |
| **EV10** | **10** | **5** | **5** | **5** | **5** | **5** | **5** | **5** | **1** |
| **EV11** | **10** | **5** | **5** | **5** | **5** | **5** | **5** | **5** | **10** |
| **EV12** | **10** | **5** | **2** | **5** | **5** | **5** | **5** | **5** | **10** |
| **Total** | **120** | **60** | **57** | **59** | **60** | **60** | **60** | **52** | **66** |

|  | **Infection status** |
| --- | --- |
|  | *Crithidia bombi* |
|  | *Apicystis bombi* |
|  | Co-infection |
|  | No *A. bombi* or *C. bombi* detected |
|  | No data |
